# Spatial Pattern of Living Woody and Coarse Woody Debris in Warm-Temperate Broad-Leaved Secondary Forest in North China

**DOI:** 10.3390/plants13162339

**Published:** 2024-08-22

**Authors:** Fang Ma, Shunzhong Wang, Weiguo Sang, Keming Ma

**Affiliations:** 1 State Key Laboratory of Urban and Regional Ecology, Research Center for Eco-Environmental Sciences, Chinese Academy of Sciences, Beijing 100085, China; 2 State Key Laboratory of Vegetation and Environmental Change, Institute of Botany, Chinese Academy of Sciences, Beijing 100093, China; 3 College of Life and Environmental Sciences, Minzu University of China, #27 Zhongguancun South Avenue, Beijing 100081, China

**Keywords:** spatial distribution pattern, indicators, habitat heterogeneity, tree mortality, sustainable development

## Abstract

The investigation into the spatial distribution of living woody (LWD) and coarse woody debris (CWD) within forests represents a fundamental methodology for probing the inherent mechanisms governing coexistence and mortality within forest ecosystems. Here, a complete spatial randomness (CSR) null model was employed to scrutinize the spatial pattern, while canonical correspondence analysis (CCA) and the Torus-translation test (TTT) were utilized to elucidate the distribution patterns of LWD and CWD within warm-temperate deciduous broadleaf secondary forests in Dongling Mountains plot, northern China. The results reveal that both LWD and CWD exhibit an aggregated distribution as the predominant pattern in the Dongling Mountains plot, with the proportion and intensity of aggregation diminishing as spatial scale increases. Specifically, the aggregation intensity g_0–10_ demonstrates a significant negative correlation with abundance and maximum diameter at breast height (DBH). Notably, the g_0–10_ of LWD manifests a stronger correlation with the maximum DBH, whereas the g_0–10_ of CWD exhibits a greater association with the mortality rate. CCA outcomes suggest that elevation, convexity, and aspect significantly impact LWD distribution, whereas CWD distribution shows substantial negative correlations with elevation, convexity, slope, and aspect. TTT findings indicate that ecosystems characterized by a substantial presence of LWD also display a notable prevalence of CWD. Additionally, the majority of species exhibit no habitat preference, displaying neutral habitat connections and low ecological niche differentiation within the sampled plot.

## 1. Introduction

The exploration of species distribution patterns within forest ecosystems has been a central focal point in ecological research [1]. Analyzing the species’ spatial distribution serves as a means to quantify the structure of ecological communities [2]. Furthermore, comprehending these distribution patterns allows for insights into the dynamic succession processes within the community and enables investigations into the mechanisms governing species coexistence and the underlying ecological processes within the community [3,4]. Typically, natural communities exhibit three primary spatial patterns among trees: (i) clumped or aggregated, (ii) regular or uniform, and (iii) random [5]. Clustered distributions are commonly observed in natural forests, especially in tropical environments [6,7]. The spatial arrangement of communities is influenced by a range of ecological processes, including ecological niche separation, habitat heterogeneity, diffusional limitations, intraspecific and interspecific competition, and negative density dependence [8,9,10]. These ecological processes can be broadly categorized into two distinct groups: biotic and abiotic processes.

In natural communities, especially within forest ecosystems, spatial patterns can be shaped by both biotic and abiotic processes, leaving distinct imprints [11]. Biotic impacts, such as the activities of host-specific natural enemies, can lead to targeted attacks on seeds or saplings, altering spatial patterns [12,13]. Additionally, the avoidance of conspecific seed-trees and offspring plays a role in diminishing the clustering of mature trees [14]. Furthermore, the impact of density-dependent intraspecific resource competition on population spatial dynamics becomes notable when resource availability drops below a limiting threshold [15]. The process of density-dependent self-thinning triggers a decline in the level of the spatial clustering of species as they mature [15]. Diffusion limitation stands out as an important biological process contributing to the comprehension of species aggregation patterns [16]. This phenomenon is particularly pronounced in the biodiverse tropics [16,17,18,19,20], where there exists a strong correlation between aggregation degree and the dispersal capacity of species [3]. Additionally, it is noteworthy that functional properties play a crucial role in impacting the spatial distribution patterns of forest species [3,6].

Regarding abiotic variables, multiple studies have demonstrated the substantial impact of habitat and topography on the spatial distribution of species [21,22]. Simultaneously, the impacts of habitat heterogeneity on species distributions may vary across different life history stages [23]. Several studies have highlighted significant positive or negative correlations between species distribution and topographical features such as slope, elevation, or aspect [24,25]. However, the ecological processes responsible for maintaining species distribution remain subjects of intense debate and have yet to be definitively addressed. This challenge is particularly evident when considering potential variations in influencing mechanisms across different forest types.

Tree mortality is a pivotal aspect of forest dynamics [26,27], significantly impacting the successional trajectory of forest communities by creating gaps for regeneration [28,29], altering carbon and nutrient cycling, and promoting tree species coexistence [30]. Tree mortality can result from various factors, including reduced space for species due to competition, natural disasters, physiological aging, and decreased species resilience caused by habitat changes. A thorough understanding of the spatial patterns and driving variables influencing tree mortality is essential for accurately predicting forest dynamics [31]. Previous research has highlighted a positive correlation between tree abundance and their preferred habitats [32], as well as higher tree mortality rates in unsuitable habitats [31]. Numerous investigations suggest that topographical features, such as elevation, convexity, slope, and aspect, play a pivotal role as abiotic environmental factors influencing the distribution, growth, and mortality of trees. This influence is primarily mediated through its effects on soil nutrients, light availability, and precipitation patterns [33]. Additionally, tree mortality is influenced not only by the surrounding environment but also by the inherent traits of the trees themselves. The size of a tree is an important trait that has been extensively examined, revealing its substantial impact on tree mortality [34]. Ganey and Vojta discovered that tree death exhibited a non-random pattern in relation to tree size class [35]. In recent years, the field of metabolic ecology has put out a theoretical proposition indicating that the rate of tree mortality decreases as tree size increases. Furthermore, it posits that larger trees possess an asymmetrical advantage over smaller trees in the context of resource competition [36]. Nevertheless, what is the manner in which coarse woody debris is dispersed across forest ecosystems? What are the key determinants that exert a major influence on the spatial distribution of coarse woody debris? These fundamental questions lack consistent and unambiguous answers.

To date, the majority of studies have focused on living woody debris (LWD) as the primary subject of investigation. Consequently, the formation process and distribution of dead wood have been inferred from examining the modifications in the structural characteristics of LWD. Yet it is important to acknowledge that the analysis of patterns can often overlook the direct usage of dead wood as a research subject. By directly examining dead wood, researchers can obtain a more accurate understanding of its formation trends, thereby avoiding potential errors that may arise from indirectly inferring information from living wood. This approach is particularly significant in uncovering the dynamic process of change at the community level. Additionally, significant basic research has been conducted in recent decades, primarily focusing on tropical rainforests, to explore mechanisms or processes that may influence spatial distribution [1,6,37,38], and subtropical forests [39,40], with few studies featuring temperate [3] and warm-temperate forests [41]. Warm-temperate forests represent a significant vegetation type in China, characterized by distinct geographic features and abundant plant resources. Beijing is located in North China and is the capital; the surrounding montane forests are primarily warm-temperate deciduous broadleaf secondary forests that have regenerated following past disturbances. A valuable platform for studying the spatial distribution and biodiversity conservation of warm-temperate forests is the 20-hectare warm-temperate dynamic monitoring sample plot located in the Dongling Mountains (DLM). In this sample site, LWD and coarse woody debris (CWD) were used for addressing the following research points: (1) to analyze whether living woody and coarse woody debris exhibit spatially nonrandom distributions in warm-temperate forests; (2) to examine whether species attributes (e.g., species abundance, diameter at breast height (DBH) class, etc.) are related to their (living woody and coarse woody debris) spatial distribution patterns; and (3) to examine whether or not habitat heterogeneity has an influence on the living woody and coarse woody debris distribution patterns of species in the plot and speculate on the cause of tree death. We will acquire a greater awareness of species coexistence and diversity conservation in warm-temperate deciduous broad-leaved secondary forests in light of this study.

## 2. Methods

### 2.1. Study Site

The study was conducted within the Beijing Xiaolongmen Forest Park Reserve (39°48′34″–40°10′37″ N, 115°25′–116°10′07″ E). The vegetation in this area comprises a typical warm-temperate deciduous broad-leaved forest, characterized by relatively complex community structures. The region experiences a warm-temperate continental monsoon climate with four distinct seasons. The mean annual temperature is 4.8 °C, with an average July (hottest) temperature of 18.3 °C, and an average January (coldest) temperature of −10.1 °C. The annual frost-free period lasts approximately 195 days, and the area receives about 2600 h of sunshine annually. Annual precipitation ranges between 500 and 650 mm, with June and August contributing approximately 78% of the total precipitation. The bedrock is Jurassic andesite. The parent soil material is mountain brown soil [42].

**Figure 1:**
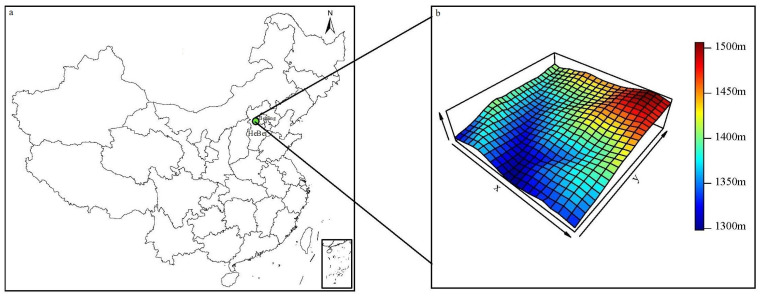
Plot location and elevation distribution schematic. Note: Image (**a**) shows plot location; (**b**) shows elevation distribution of each sample plot.

A 20 ha (400 m × 500 m) plot was established in 2010 in Xiaolongmen Forest Park Reserve at coordinates 40°00′ N, 115°26′ E. The initial census was conducted in 2010 following the standard field protocol of the CTFS (Center for Tropic Forest Sciences, http://www.ctfs.si.edu). The plot is situated in rugged terrain (Figure 1), with elevations ranging from 1298.21 m to 1506.34 m and slopes varying from 8.46° to 48.49°, with a mean slope of 31.98°.

### 2.2. Data Collection

The 20-hectare plot, divided into 500 subplots measuring 20 × 20 m^2^ each, was surveyed using standard field methods to identify, tag, and map all free-standing trees with a diameter at breast height (DBH) of at least 1 cm [43]. A comprehensive survey of living plants across the 500 plots resulted in the identification of 56 species, 36 genera, and 20 families. Coarse woody debris with a DBH greater than or equal to 5 cm was also identified, tagged, and mapped based on the living dataset. In total, 32 species, 25 genera, and 15 families of coarse woody debris were recorded (unidentified species labeled as “unknown”). Acer mono Maxim and Quercus wutaishanica emerged as the dominant species in our plot. To ensure an adequate sample size for point pattern analyses, we selected 46 common species, with a minimum of 20 individuals each [44].

### 2.3. Data Analyses

Spatial point pattern analysis has been widely utilized to examine the spatial distribution patterns of various species [45]. In this study, the function g(r) was employed to investigate the spatial distribution of species within a range of 0–50 m. Furthermore, the mean g_0–10_ was used as a metric to quantify the average conspecific aggregation density within a 10 m radius of a tree, following methods outlined in previous publications [6]. The g function is based on Ripley’s K function [46]. The Ripley’s K function is a cumulative distribution function; however, the g function, which is a non-cumulative distribution function and a probability density function, presents the expected density of trees in a circular ring of a given distance r around a focal tree, divided by the intensity of the pattern, effectively eliminating the cumulative effect of the Ripley’s K function as the scale increases. This density is then normalized by the overall intensity of the pattern. In our study, complete spatial randomness (CSR) was used as the null model, suggesting that tree occurrences are independent of biological processes. To test whether a species deviates significantly from the random distribution, we conducted 999 Monte Carlo simulations. If the observed values fall within the 2.5th and 97.5th percentiles, the null model cannot be rejected, indicating that the species is aggregated. These simulations were conducted using the “spatstat” package in R.

The mean aggregation intensity g_0–10_ was employed to evaluate the distribution patterns of both LWD and CWD. Analysis revealed that the g_0–10_ values across all species deviated from normality. Therefore, Spearman’s correlation analysis was conducted to explore the relationship between various species traits and population aggregation intensity. Species abundance was categorized into three levels: abundant (≥1000 individuals per 20 ha), common (100–999 individuals/20 ha), and rare (<100 individuals/20 ha). The statistical significance of the data was determined using either the Kruskal–Wallis or Wilcoxon rank sum test. Furthermore, the identified species were classified into three dispersal modes: animal, gravity, and wind.

**Figure 2:**
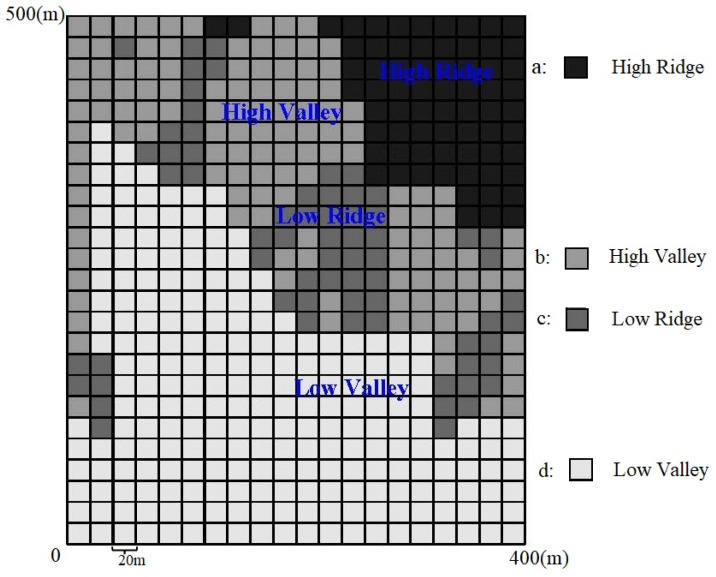
The 500 20 × 20 m quadrats of 20 hm^2^ plots divided into four habitat types.

The topographic variables, including elevation, slope, aspect, and convexity, were selected to explore species–habitat associations within each 20 × 20 m grid. The researchers employed the MRT (Multivariate Regression Tree) method [47] to classify the 500 cells into four distinct habitat types: high ridge, low ridge, high valley, and low valley. This analysis was conducted using the “rpart” package in R (Figure 2).

Canonical Correlation Analysis (CCA) was used to examine the correlation between topography (including elevation, slope, aspect, and convexity) and the spatial distribution of woody vegetation at the community level. To evaluate the significance of these relationships, a Monte Carlo permutation test was conducted using the “vegan” package in R. Each topographic variable underwent 1000 random permutations, with a significance level of 5%. All statistical analyses were performed using R version 4.3.1 (R Development Core Team) and Microsoft Excel 2023.

There exist various methodologies that can be employed to examine the association between species and habitats. Due to the presence of auto-correlation in species distributions, we opted to utilize the Torus-translation test (TTT) instead of applying conventional measurements. A comprehensive account of the Torus-translation test (TTT) can be located in the study conducted by Harms [8].

## 3. Result

### 3.1. General Spatial Pattern

**Figure 3:**
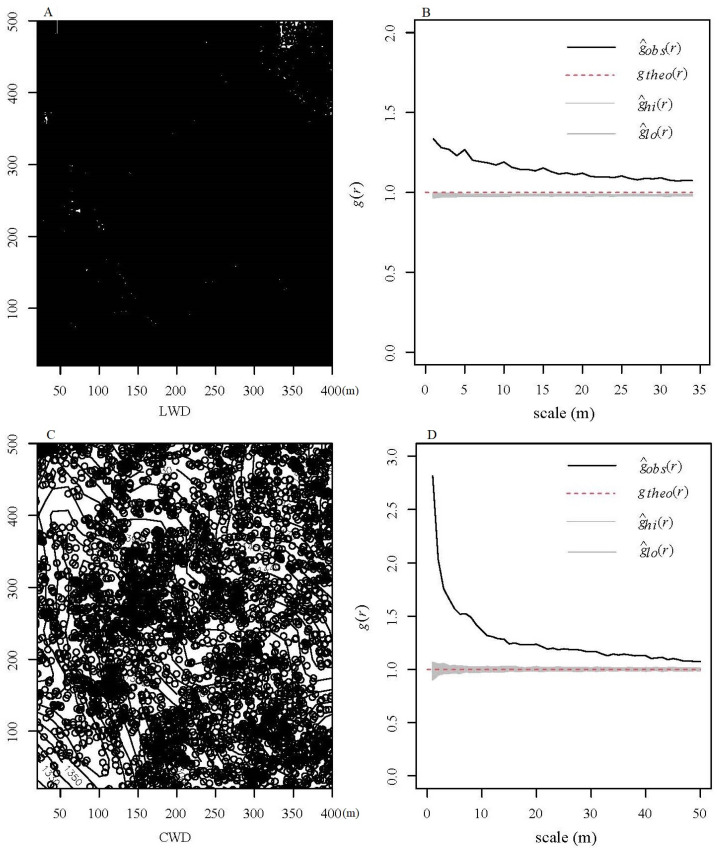
Spatial distribution of living woody (LWD) and coarse woody debris (CWD). Images (**A**,**C**) show corresponding distribution patterns. (**B**,**D**) show relationship between univariate pair-correlation function (g(r)).

**Figure 4:**
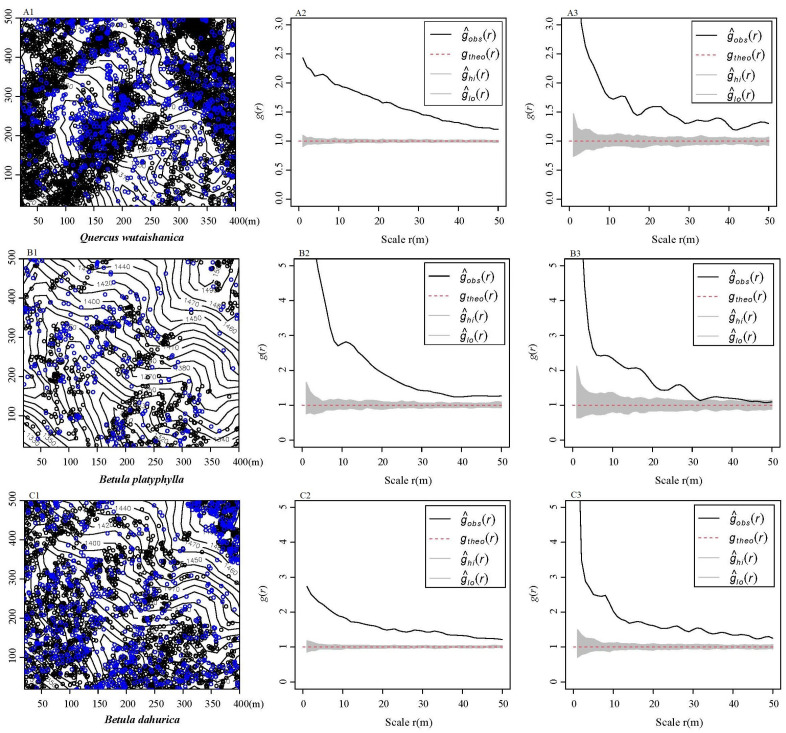
Examples of the spatial distribution patterns of the three dominant species. Note: images (**A1**,**B1**,**C1**) display the distribution of each of the three species across the terrain, and the black dots represent living woody (LWD), the blue dots represent coarse woody debris (CWD). (**A2**,**B2**,**C2**) show the distribution of the three living woody debris (LWD) species, while (**A3**,**B3**,**C3**) show the distribution of the three coarse woody debris (CWD) species.

The analysis focused on examining the spatial pattern of 46 species of LWD and 22 species of CWD within a range of 0 to 50 m from the DLM sample site. This investigation utilized a CSR null model. The study revealed that all of the species under consideration exhibited aggregation patterns at a smaller scale (Figure 3). Moreover, as the spatial scales expanded, the fraction of aggregated species declined, while the proportions of random and regular patterns increased. A total of 46 species (100%) of LWD were aggregated at a scale of 23 m, 45 species were aggregated at a scale of 24 to 27 m, and 40 species (86.96%) were aggregated at a scale of 50 m. The proportion of aggregated CWD declined to 54.55% at 50 m. Furthermore, the aggregation intensity g_0–10_(r) for both LWD and CWD showed a gradual decline as spatial scales increased. The aggregation intensity of LWD was smaller in comparison to CWD. Using the spatial distribution of the three dominant species as a case study, the populations of various species were aggregated into distinct habitats (Figure 4).

### 3.2. Attributes and Distribution Patterns

**Figure 5:**
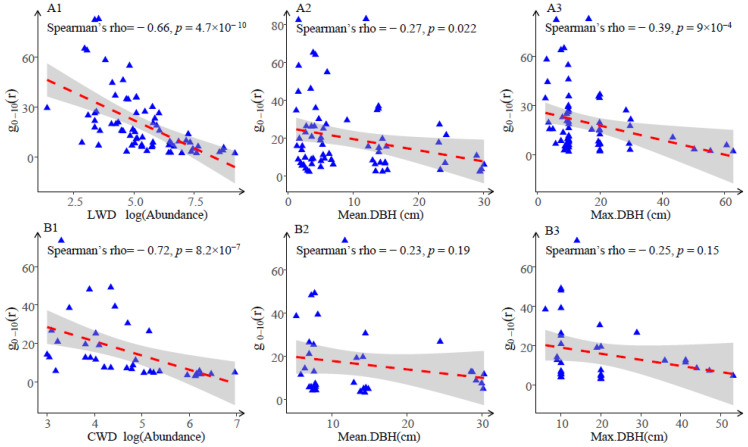
Relationship between aggregation index (g_0–10_(r)) and abundance, mean DBH, and maximum DBH of living woody and coarse woody debris. Note: images (**A1**,**A2**,**A3**) show living woody (LWD); (**B1**,**B2**,**B3**) show coarse woody debris (CWD).

According to the CSR null model, there was a negative relationship seen between the aggregation intensity (g_0–10_) and the variables of species abundance, mean diameter, and maximum DBH (Figure 5). The correlation between LWD and CWD abundance and g_0–10_ was a significant negative correlation (LWD: Spearman’s rho = −0.66, *p* < 0.001; CWD: Spearman’s rho = −0.72, *p* < 0.001), the maximum DBH and g_0–10_ had a significant negative association (LWD: Spearman’s rho = −0.39, *p* < 0.001; CWD: Spearman’s rho = −0.25, *p* < 0.05), the mean DBH and g_0–10_ had a significant negative link (LWD: Spearman’s rho = −0.27, *p* < 0.05), and there was a non-significant link observed between the mean DBH of CWD and g_0–10_.

There is a strong association between the maximum DBH of LWD and g_0–10_, and a significant correlation between the abundance of CWD and g_0–10_. It is postulated that the spatial distribution of LWD is determined by the presence of large trees, while the CWD is influenced by mortality rates.

The results of the Kruskal–Wallis test revealed statistically significant variations in the aggregation intensity g_0–10_ among the three different abundance levels of LWD (χ2 = 36.95, *p* < 0.001). The g_0–10_ of the abundant species was significantly lower compared to both the common and rare species. The sampling of rare species of CWD was insufficient for the purpose of comparison. This finding corresponds to the result that the g_0–10_ declines as abundance increases, and the fluctuation of g_0–10_ diminishes as the amount of individuals increases. Furthermore, the outcomes of the Kruskal–Wallis test indicated that there was non-significant variance in the aggregation intensity g_0–10_ across different dispersal strategies (LWD: χ2= 4.12, *p* = 0.25; CWD: χ2= 3.07, *p* = 0.22).

### 3.3. Topographic Variables and Habitat Heterogeneity

**Figure 6:**
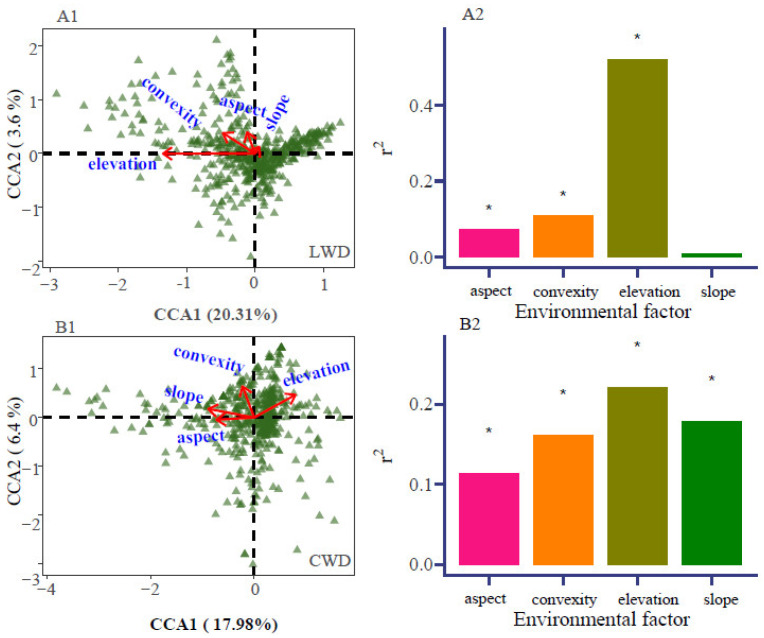
Canonical correspondence analysis (CCA) biplot of living woody and coarse woody debris across topographic factors. Note: images (**A1**,**A2**) show living woody debris (LWD); (**B1**,**B2**) show coarse woody debris (CWD). * represents the significance of each environmental factor’s impact.

The results demonstrated that the four topographic parameters had a significant impact on species distribution. Nevertheless, the effects of topographic factors on the distribution of LWD and CWD were different. The spatial distribution of LWD exhibited a substantial negative correlation with elevation, convexity, and aspect (*p* < 0.001), but a non-significant association was seen with slope (Table 1). The pattern of CWD was strongly influenced by elevation, convexity, aspect, and slope (*p* < 0.01) (Table 1). The spatial distributions of living woody plants were significantly negatively correlated with elevation, convexity, and aspect on the first axis, but positively correlated with slope. On the second axis, they were negatively correlated with elevation and positively correlated with the other three topographic factors. In contrast, the spatial distributions of coarse woody debris were negatively correlated with slope, convexity, and aspect on the first axis, but positively correlated with elevation. On the second axis, they were negatively correlated with aspect and positively correlated with the other three topographic factors (Figure 6).

**Figure 7:**
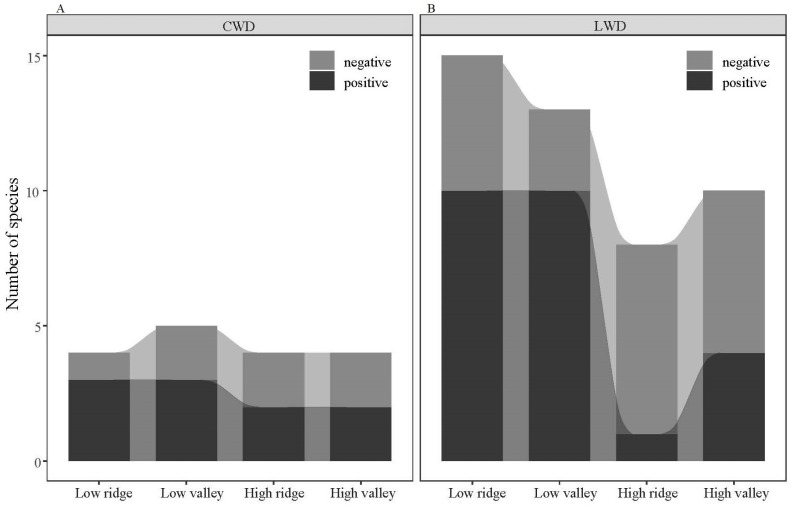
Habitat associations of living woody and coarse woody debris across different topographic factors. Note: image (**A**) shows coarse woody debris (CWD); (**B**) shows living woody debris (LWD).

According to the findings of the Torus-translation test (Figure 7), in the case of LWD, there were 20 (43.48%) species significantly positively associated with the low ridge and valley, and 4 and 1 species significantly positively associated with the high valley and ridge. There was a negative correlation observed between seven species and high ridges, while three species exhibit a negative correlation with low valleys. *Betula platyphylla* Suk. demonstrates a positive association with low ridges while displaying a negative association with low valleys. This can likely be attributed to the varying habitat preferences across different life history stages of the species. The majority of species exhibited a neutral status, meaning they did not display significant habitat preferences or repulsion for any of the four habitats.

Most species of CWD exhibited a positive correlation with the low valley, while the high valley and ridge displayed a comparatively smaller number of species. Some species showed both positive and negative correlations with specific habitats, such as the Fraxinus chinensis subsp., which showed a positive association with low valleys while presenting a negative correlation with high ridges. Acer mono Maxim. had a significant positive link with low and high ridges, suggesting that the species experiences increased mortality in both habitats. The majority of species demonstrate neither preference nor repulsion towards their habitat and can potentially become abundant or common within their own ecosystems.

Furthermore, the observed distribution pattern of positive and negative correlations indicates that the scale of the various habitats did not have a statistically significant impact on the correlations.

## 4. Discussion

### 4.1. Spatial Pattern of Woody Plants

The study revealed that the woody vegetation exhibited a clustered distribution pattern at the local scale in Dongling Mountains. This finding further supports the inference that the majority of species exhibit an aggregated distribution; only a small number of species display a random and regular spatial pattern [3,6]. The present study reveals that LWD and CWD showed an aggregated spatial distribution and corresponds to previous research conducted on the spatial pattern found in tropical, subtropical, and temperate forests [3,39,48], following the regularity of population distribution in natural communities. Community patterns have an obvious scale-dependent effect, whereby the distribution of species demonstrates variability across different scales [9]. This study also found a steady decline in the percentage of aggregated species and the aggregation intensity of LWD and CWD at the sample site as scales increased, indicating the existence of scale-dependent effects.

The predominant spatial pattern of LWD is known to be an aggregated distribution. It has been proposed that the spatial distribution may be influenced by localized dispersal centered on propagules, as well as dispersal limitations. The seed quantity decreasing as distance from the parent plant increases, along with the variations in seed production during dispersal, can be seen as an indirect confirmation of some predictions put forth by the Janzen–Connell hypothesis [12,13]. Likewise, among tropical forest ecosystems, the density of neighboring density shows a substantial decrease as the distance increases [6]. This implies that dispersal limitations have a significant impact on the spatial distribution of species at the local scale. Furthermore, the patchy allocation of resources such as water, light, and soil nutrients, resulting in the formation of diverse habitats, can also exert an effect on the species distribution [49,50,51].

The investigation utilized spatial point pattern analysis to examine the spatial randomness of tree death, and the association between LWD and CWD was studied to assess the potential influence of intraspecific or interspecific competition [52,53]. The results of our research indicate that the distribution of CWD in the warm-temperate forest exhibits typical clustering patterns within a range of 0–50 m. This suggests that the occurrence of spatial mortality in this forest is not random (Figure 1). The occurrence of non-randomized tree mortality patterns is frequently seen in forest ecosystems [30,53], and usually occurs due to the inherent traits of species and the current habitat [31], allowing for the rejection of the random death hypothesis.

### 4.2. Attributes of Spatial Distribution

Prior research has demonstrated that the spatial distribution of species is affected by functional traits such as species diversity, size structure, and dispersion strategy [44]. The current study found a significant negative correlation between the aggregation intensity g_0–10_ and both abundance and max (mean) diameter of LWD and CWD. These results provide further support for the conclusions obtained in prior studies [10]. The aggregation intensity of rare species in our sample was greater in comparison to abundant and common species; this finding coincides with previous studies conducted in other forest ecosystems [9,10,44], and may be attributed to the pronounced habitat preference exhibited by extremely small populations. The decline in aggregation intensity as the maximum diameter class increases is primarily caused by self-thinning [39,48]. Simultaneously, herbivores and pests might also assist in the reduction in aggregation [8].

Additionally, dispersal strategies are acknowledged as possible approaches to accomplish spatial separation among species and mitigate competitive exclusion, and the species aggregation intensity is highly connected with the capacity to disperse seeds [3,54]. Species that depend on animal dispersal are commonly regarded as possessing a higher dispersal capacity compared to those distributed through gravity and wind [6].

Nevertheless, it has also been suggested that the differences seen across the three dispersal modes lacked significance [10], a finding that aligns with our own research. Based on the aforementioned evidence, it is postulated that the species-abundance relationship could potentially serve as a fundamental factor in several ecological processes, including but not limited to diffusion limitation that governs spatial patterns. It is imperative to conduct further research in order to substantiate this claim.

The results of this study showed an association between tree attributes, and the aggregation intensity was consistent both in LWD and CWD. Regarding the alterations of forest trees pre- and post-mortality, the aggregation intensity will experience a reduction and lead to a more regular distribution if the event of density-dependent mortality is observed [55]. An investigation exploring variations in spatial distributions of forest species pre- and post-mortality revealed that there exists a density-dependent mortality in temperate old-growth coniferous forests [55].

The conclusions of this study suggest that there is a significant aggregation among small-diameter classes of LWD, indicating ecological niche overlap and intense competition during the growth process of LWD, which leads to the aggregated distribution among small-diameter classes of CWD. As the diameter class of trees increased, there was a noticeable drop in the strength of the self-thinning effect, as well as a decline in the aggregation intensity observed among both LWD and CWD. Based on the aforementioned investigation, it is possible to assume that the likely factor contributing to tree mortality in the sample is negatively density-dependent.

### 4.3. Topography and Habitat of Spatial Distribution

Topography holds importance in assessing habitat conditions and plays a crucial role in shaping community spatial distribution [56]. The topographic factor is complex and comprehensive, with its external manifestation being the result of the combined influence of characteristics such as elevation, convexity, slope, and aspect [57]. Research indicates that the spatial distribution is influenced by topography, as it plays a role in the redistribution of crucial resources such as soil nutrients, precipitation, and light [33,50,58].

The outcomes of the present study based on the results of CCA show that there exists a significant negative correlation between the spatial distribution of LWD and elevation, convexity, and aspect, with the exception of slope. The distribution of CWD was found to be strongly influenced by elevation (with the only significant positive correlation), convexity, slope, and aspect (the latter three with a significant negative correlation). In this study, it was observed that both LWD and CWD were greatly affected by elevation, which emerged as the most crucial topographic element impacting species distribution.

The distribution of living tree species in the Xishuangbanna Tropical Rainforest Dynamic Monitoring Sample Plot was primarily influenced by altitude [38]; with the subtropical evergreen broad-leaved mixed forests in Badagong Mountains, only altitude exhibited a statistically significant impact on tree mortality [38]. Topographic factors are indirect ecological factors that exert an influence on spatial patterns, and tree mortality could potentially be impacted by covariation with environmental conditions.

The spatial distribution patterns of woody plants at large scales are affected by habitat heterogeneity, which is widely acknowledged as an important process of governing aggregation patterns [38,59]. The distribution and survival, or mortality, of species are greatly impacted by their habitat, with some species exhibiting competitive advantages in their preferred habitat [32]; conversely, when species are in unfavorable habitats, mortality rates are likely to increase [3]. The results of the TTT show that not all species exhibit specific habitat preferences, a conclusion that correlates with previous studies [10,25,60]. The distribution patterns of species may be influenced by various habitats, which can affect the redistribution of light, temperature, soil moisture, and nutrients [9].

The findings of this study indicate a positive correlation between LWD and low ridges as well as low valleys, while a negative association exists between LWD and high ridges as well as high valleys. Similarly, CWD showed positive correlations with low valleys and ridges, mirroring the pattern observed with LWD. Additionally, CWD displayed nearly identical negative links with the four habitat types. If LWD has a negative relationship with a particular habitat, it suggests that the species is unable to regenerate and persist in such circumstances. On the other hand, a positive or neutral association reflects the species’ ability to sustain populations in these regions. While a species might positively correlate with a particular habitat type, it generally demonstrates neutrality with the remaining three habitat types. This implies that, although species exhibit preferences for specific habitats, they are not restricted from persisting in alternative habitats. The habitat preferences for CWD correspond with LWD, showing that low ridge and valley habitats are beneficial to root development. However, (1) large populations lead to greater competition for resources, (2) valleys experience a year-round influx of water and substantial erosion, and (3) frequent human-induced disturbances at low elevations contribute to the abundance of CWD. The majority of species had a neutral inclination for habitats, and habitat associations alter with different life history phases. In conjunction with investigations into topography and habitat correlations, it was determined that ecological niche differentiation has a role in facilitating the coexistence of species, but its impact is modest, and negative density-dependent effects should be considered.

Comprehending the spatial pattern of trees within a specific area is of paramount importance for effective land management and the implementation of silvicultural strategies that emulate natural conditions. Consequently, managerial decisions should be executed with careful consideration for the primary characteristics of both LWD and CWD within the forested region. The principal objectives of these interventions aim to optimize the spatial distribution and composition of the forest, prioritize the establishment of regeneration, and focus efforts on the conservation of the overall forest ecosystem.

## 5. Conclusions

Based on our research findings, we present instances of silvicultural measures applicable to both LWD and CWD. In the initial phase, when self-thinning emerges as the predominant mechanism among lower and middle-tier trees, the implementation of tree thinning can expedite this inherent process and enhance tree diversity. Commercial exploitation can be employed to promote the growth in diameter of dominant trees, capitalizing on their structural attributes through effective management at the opportune developmental stage. During the decay phase, the practice of sanitary felling can be applied to remove fallen wood, mitigating the potential harboring of pests and diseases, and concurrently safeguarding the regeneration of trees.

This study presents a comprehensive analysis of the spatial distribution of species in warm-temperate deciduous broad-leaved secondary forests in China. The findings indicated that both LWD and CWD were predominantly found in clusters, and tree mortality did not occur randomly. The distribution patterns of LWD and CWD were driven by a combination of their inherent traits and habitat heterogeneity. The consideration of negative density dependence is crucial when examining the mechanisms of species coexistence in sample plots. The results of this study offer significant information about the management of the spatial distribution pattern in warm-temperate secondary forest. Additionally, they serve as a valuable reference for promoting the sustainable exploitation of regional forests.

## Figures and Tables

**Table 1:** The permutation test for the topographic factors explaining the distributions of living woody and coarse woody debris.

Living Woody Debris	CCA1	CCA2	R^2^	Pr (>r)
Elevation	−0.996	−0.088	0.521	0.001 ***
Convex	−0.872	0.489	0.109	0.001 ***
Slope	0.693	0.721	0.009	0.106
Aspect	−0.126	0.992	0.072	0.001 ***
Coarse Woody Debris	CCA1	CCA2	R^2^	Pr (>r)
Elevation	0.853	0.521	0.221	0.001 ***
Convex	−0.306	0.952	0.162	0.001 ***
Slope	−0.981	0.195	0.178	0.001 ***
Aspect	−0.997	−0.077	0.113	0.001 ***

Note: *** *p* < 0.001. *p* is the result of 1000 permutations of the test; R^2^ is the determination coefficient of topographic factors; Pr represents a significance test of correlation.

## Data Availability

Data are contained within the article.

## References

[1] Wiegand T., Gunatilleke S., Gunatilleke N., Okuda T. (2007). Analyzing the spatial structure of a sri lankan tree species with multiple scales of clustering. Ecology.

[2] Perry G., Enright N.J., Miller B.P., Lamont B.B. (2009). Nearest-neighbor interactions in species-rich shrublands: The roles of abundance, spatial patterns and resources. Oikos.

[3] Wang X., Ye J., Li B., Zhang J., Lin F., Hao Z. (2010). Spatial distributions of species in an old-growth temperate forest, northeastern China. Can. J. For. Res..

[4] Wiegand T., Uriarte M., Kraft N.J., Shen G., Wang X., He F. (2017). Spatially explicit metrics of species diversity, functional diversity, and phylogenetic diversity: Insights into plant community assembly processes. Annu. Rev. Ecol. Evol. Syst..

[5] Larsary M.K., Abkenar K.T., Pourbabaei H., Pothier D., Amanzadeh B. (2018). Spatial patterns of trees from different development stages in mixed temperate forest in the hyrcanian region of Iran. J. For. Sci..

[6] CCondit R., Ashton P.S., Baker P., Bunyavejchewin S., Gunatilleke S., Gunatilleke N., Hubbell S.P., Foster R.B., Itoh A., LaFrankie J.V. (2000). Spatial patterns in the distribution of tropical tree species. Science.

[7] Hai N.H., Wiegand K., Getzin S. (2014). Spatial distributions of tropical tree species in northern Vietnam under environmentally variable site conditions. J. For. Res..

[8] Harms K.E., Condit R., Hubbell S.P., Foster R.B. (2001). Habitat associations of trees and shrubs in a 50 ha neotropical forest plot. J. Ecol..

[9] Du H., Hu F., Zeng F., Wang K., Peng W., Zhang H., Zeng Z., Zhang F., Song T. (2017). Spatial distribution of tree species in evergreen-deciduous broadleaf karst forests in southwest China. Sci. Rep..

[10] Song H., Xu Y., Hao J., Zhao B., Guo D., Shao H. (2017). Investigating distribution pattern of species in a warm-temperate conifer-broadleaved-mixed forest in China for sustainably utilizing forest and soils. Sci. Total Environ..

[11] Hubbell S.P., Ahumada J.A., Condit R., Foster R.B. (2001). Local neighborhood effects on long-term survival of individual trees in a neotropical forest. Ecol. Res..

[12] Janzen D.H. (1970). Herbivores and the number of tree species in tropical forests. Am. Nat..

[13] Connell J.H. (1971). On the role of natural enemies in preventing competitive exclusion in some marine animals and in rain forest trees. Dyn. Popul..

[14] Hamill D.N., Wright S.J. (1986). Testing the dispersion of juveniles relative to adults: A new analytic method: Ecological archives e067-004. Ecology.

[15] Kenkel N.C. (1988). Pattern of self-thinning in jack pine: Testing the random mortality hypothesis. Ecology.

[16] Scherrer D., Körner C. (2011). Topographically controlled thermal-habitat differentiation buffers alpine plant diversity against climate warming. J. Biogeogr..

[17] Hubbell S.P. (2008). Approaching ecological complexity from the perspective of symmetric neutral theory. Trop. For. Community Ecol..

[18] Dalling J.W., Muller Landau H.C., Wright S.J., Hubbell S.P. (2002). Role of dispersal in the recruitment limitation of neotropical pioneer species. J. Ecol..

[19] Wright J.S. (2002). Plant diversity in tropical forests: A review of mechanisms of species coexistence. Oecologia.

[20] Muller-Landau H.C., Wright S.J., Calderón O., Condit R., Hubbell S.P. (2008). Interspecific variation in primary seed dispersal in a tropical forest. J. Ecol..

[21] Gunatilleke C.V.S., Gunatilleke I.A.U.N., Esufali S., Harms K.E., Ashton P.M.S., Burslem D.F.R.P. (2006). Species-habitat associations in a sri lankan dipterocarp forest. J. Trop. Ecol..

[22] Queenborough S.A., Burslem D.F., Garwood N.C., Valencia R. (2007). Habitat niche partitioning by 16 species of myristicaceae in Amazonian Ecuador. Plant Ecol..

[23] Shen G., He F., Waagepetersen R., Sun I.-F., Hao Z., Chen Z.-S., Yu M. (2013). Quantifying effects of habitat heterogeneity and other clustering processes on spatial distributions of tree species. Ecology.

[24] Svenning J. (2001). Environmental heterogeneity, recruitment limitation and the mesoscale distribution of palms in a tropical montane rain forest (maquipucuna, Ecuador). J. Trop. Ecol..

[25] Ye J., Hao Z., Xie P., Li J. (2011). Habitat associations of saplings and adults in an old-growth temperate forest in the changbai mountains, northeastern China. For. Stud. China.

[26] Franklin J.F., Shugart H.H., Harmon M.E. (1987). Tree death as an ecological process. Bioscience.

[27] Hilger A.B., Shaw C.H., Metsaranta J.M., Kurz W.A. (2012). Estimation of snag carbon transfer rates by ecozone and lead species for forests in Canada. Ecol. Appl..

[28] Rüger N., Huth A., Hubbell S.P., Condit R. (2011). Determinants of mortality across a tropical lowland rainforest community. Oikos.

[29] Song H., Zhou D., Chen S., Li J., Wang C., Ren Y., Yang X. (2023). Disparity in the relative roles of biotic and abiotic drivers on tree mortality between warm-temperate and temperate forests in China. For. Ecol. Manag..

[30] Wu H., Franklin S.B., Liu J., Lu Z. (2017). Relative importance of density dependence and topography on tree mortality in a subtropical mountain forest. For. Ecol. Manag..

[31] Wang X., Comita L.S., Hao Z., Davies S.J., Ye J., Lin F., Yuan Z. (2012). Local-scale drivers of tree survival in a temperate forest. PLoS ONE.

[32] Tilman D., Pacala S. (1993). The maintenance of species richness in plant communities. Scanning Electron Microsc Meet.

[33] Zhang L., Mi X., Shao H., Ma K. (2011). Strong plant-soil associations in a heterogeneous subtropical broad-leaved forest. Plant Soil.

[34] He C., Jia S., Luo Y., Hao Z., Yin Q. (2022). Spatial distribution and species association of dominant tree species in huangguan plot of qinling mountains, China. Forests.

[35] Ganey J.L., Vojta S.C. (2011). Tree mortality in drought-stressed mixed-conifer and ponderosa pine forests, Arizona, USA. For. Ecol. Manag..

[36] Coomes D.A. (2006). Challenges to the generality of WBE theory. Trends Ecol. Evol..

[37] Kubota Y., Kubo H., Shimatani K. (2007). Spatial pattern dynamics over 10 years in a conifer/broadleaved forest, northern Japan. Plant Ecol..

[38] Lan G., Getzin S., Wiegand T., Hu Y., Xie G., Zhu H., Cao M. (2012). Spatial distribution and interspecific associations of tree species in a tropical seasonal rain forest of China. PLoS ONE.

[39] Li L., Huang Z., Ye W., Cao H., Wei S., Wang Z., Lian J., Sun I., Ma K., He F. (2009). Spatial distributions of tree species in a subtropical forest of China. Oikos.

[40] Luo Z., Ding B., Mi X., Yu J., Wu Y. (2009). Distribution patterns of tree species in an evergreen broadleaved forest in eastern China. Front. Biol. China.

[41] Piao T., Comita L.S., Jin G., Kim J.H. (2013). Density dependence across multiple life stages in a temperate old-growth forest of northeast China. Oecologia.

[42] Ma F., Zhuang L., Wang S., Sang W. (2021). Coarse woody debris features of a warm temperate deciduous broad-leaved forest, northern China. J. For. Res..

[43] Condit R. (1998). Tropical Forest Census Plots: Methods and Results from Barro Colorado Island, Panama and a Comparison with Other Plots.

[44] Guo Y., Lu J., Franklin S.B., Wang Q., Xu Y., Zhang K., Bao D., Qiao X., Huang H., Lu Z. (2013). Spatial distribution of tree species in a species-rich subtropical mountain forest in central China. Can. J. For. Res..

[45] Wiegand T., A Moloney K. (2004). Rings, circles, and null-models for point pattern analysis in ecology. Oikos.

[46] Illian J., Penttinen A., Stoyan H., Stoyan D. (2008). Statistical Analysis and Modelling of Spatial Point Patterns.

[47] De’Ath G. (2002). Multivariate regression trees: A new technique for modeling species–environment relationships. Ecology.

[48] Nguyen H.H., Uria Diez J., Wiegand K. (2016). Spatial distribution and association patterns in a tropical evergreen broad-leaved forest of north-central Vietnam. J. Veg. Sci..

[49] Wright S.J., Muller-Landau H.C., Condit R., Hubbell S.P. (2003). Gap-dependent recruitment, realized vital rates, and size distributions of tropical trees. Ecology.

[50] Engelbrecht B.M.J., Comita L.S., Condit R., Kursar T.A., Tyree M.T., Turner B.L., Hubbell S.P. (2007). Drought sensitivity shapes species distribution patterns in tropical forests. Nature.

[51] Mangan S.A., Schnitzer S.A., Herre E.A., Mack K.M.L., Valencia M.C., Sanchez E.I., Bever J.D. (2010). Negative plant–soil feedback predicts tree-species relative abundance in a tropical forest. Nature.

[52] Petritan I.C., Marzano R., Petritan A.M., Lingua E. (2014). Overstory succession in a mixed quercus petraea–fagus sylvatica old growth forest revealed through the spatial pattern of competition and mortality. For. Ecol. Manag..

[53] Lutz J.A., Larson A.J., Furniss T.J., Donato D.C., Freund J.A., Swanson M.E., Bible K.J., Chen J., Franklin J.F. (2014). Spatially nonrandom tree mortality and ingrowth maintain equilibrium pattern in an old-growth pseudotsuga-tsuga forest. Ecology.

[54] Seidler T.G., Plotkin J.B. (2006). Seed dispersal and spatial pattern in tropical trees. PLoS Biol..

[55] Getzin S., Dean C., He F., Trofymow J.A., Wiegand K., Wiegand T. (2006). Spatial patterns and competition of tree species in a douglas fir chronosequence on vancouver island. Ecography.

[56] Comita L.S., Uriarte M., Thompson J., Jonckheere I., Canham C.D., Zimmerman J.K. (2009). Abiotic and biotic drivers of seedling survival in a hurricane-impacted tropical forest. J. Ecol..

[57] Randin C.F., Engler R., Normand S., Zappa M., Zimmermann N.E., Pearman P.B., Vittoz P., Thuiller W., Guisan A. (2009). Climate change and plant distribution: Local models predict high-elevation persistence. Glob. Change Biol..

[58] Fladeland M.M., Ashton M.S., Lee X. (2003). Landscape variations in understory par for a mixed deciduous forest in new England, USA. Agric. For. Meteorol..

[59] Yue K., Yang W., Peng C., Peng Y., Zhang C., Huang C., Tan Y., Wu F. (2016). Foliar litter decomposition in an alpine forest meta-ecosystem on the eastern Tibetan plateau. Sci. Total Environ..

[60] Valencia R., Foster R.B., Villa G., Condit R., Svenning J., Hernández C., Romoleroux K., Losos E., Magård E., Balslev H. (2004). Tree species distributions and local habitat variation in the amazon: Large forest plot in eastern Ecuador. J. Ecol..

